# What Is on Your Gig Radar? Toward a Hierarchical Structure of Coping

**DOI:** 10.3390/ijerph192114219

**Published:** 2022-10-31

**Authors:** Samira A. Sariraei, Denis Chênevert, Christian Vandenberghe

**Affiliations:** 1 Human Resources Management Department, HEC Montréal, 3000 Côte–Sainte–Catherine, Montreal, QC H3T 2A7, Canada; 2 Department of Management, HEC Montréal, 3000 Côte–Sainte–Catherine, Montreal, QC H3T 2A7, Canada

**Keywords:** digital economy, gig worker, basic psychological needs, coping, adaptation, autonomy, coping structure

## Abstract

Digitalized independent workers, known as gig workers, have been shown to work under high-pressure, with a lack of autonomy, a lack of feedback and perceived competence, and a high level of isolation. We conducted a literature review to investigate how gig workers cope with these sources of stress. We identified primary sources of psychological stress in gig work and the main strategies used by workers for coping with them. We show that focusing solely on identifying coping strategies depicts a fragmented literature, making it impossible to compare, link, or aggregate findings. We suggest a radar classification of coping based on the motivational action theory of coping and self-determination theory that defines coping as a process to adapt to the environment and maintain well-being. We argue that this framework is both relevant and necessary for developing research on gig-worker coping.

## 1. Introduction

Like any species, we are hard-wired to protect ourselves from threats to health and safety to survive [1]. Threats to well-being are adaptational problems shared by animal species and humans. Yet, humans uniquely face the challenges brought to well-being by abrupt changes in technology and work forms. Our capability to design computer-programmed procedures that transform input data into desired outputs (i.e., algorithmic technologies) has created new forms of work that differ from traditional employment relationships requiring us to adapt to their challenges. For instance, Uber work, such as ride or delivery, has shown to be very helpful to societies during the COVID pandemic, but it also demonstrated the precariousness of workers and the lack of protections for them.

Researchers on new forms of work have identified “paradoxes” regarding organizational health and safety (OHS). For instance, one of the most studied yet conflicting areas of the literature on new digital forms of labor, namely, gig work, concerns the “autonomy paradox”. Employees wanting to work anytime, anywhere end up working every time and everywhere [2]. “The gig economy is an emerging labor market wherein organizations engage independent workers for short-term contracts (“gigs”) to create virtual jobs, often by connecting workers to customers via a platform-enabled digital marketplace” [3] (pp. 192–193). For instance, the Uber drive platform connects drivers (gig workers) to riders (customers), and the worker provides the service for customers under the regulations of the job designed by the Uber organization.

Gig workers’ behavior, compared to traditional employment settings, is not controlled by managerial interactions, and in theory, they can choose when to work. However, platforms enable control of these workers through regulations on how the work should be done and the penalization of workers. Platforms can collect a variety of data from workers and monitor many aspects of worker behavior, setting goals that workers usually have little freedom to accept or decline. They have little time to decide, and both their performance rate and future assignments get affected if they refuse the task [4]. Gig work structures substitute behavioral control by supervisors with technical control [5], so while it gives workers some forms of autonomy (e.g., flextime) [3], different contextual characteristics of gig work are depleting workers’ autonomy due to controls on work methods, decision making, work time through rules, ratings, penalizations, and information asymmetry [2,3,4,5,6,7,8,9,10,11,12,13]. Algorithmic control threatens worker well-being due to the ambiguity, anxiety, and insecurity resulting from these challenges [14,15]. To understand how workers respond to the demands of gig work, scholars have been interested in coping, which is generally referred to as the way individuals respond to a threat from a person–environment perspective, since it has been shown to reduce vs. amplify the effect of negative events and conditions on emotional distress and well-being [16]. In this theoretical piece, we address the challenges and threats of new forms of work, specifically gig work, for worker well-being and how workers cope to protect themselves from harm. 

We suggest that the current approach in the study of gig workers’ coping strategies will lead to a lack of consensus on coping, which impedes the development of the field, practically, theoretically, and methodologically. While the literature on gig coping is still young and growing, scholars in this field are borrowing theories from more mature streams in the literature and are following their approach to examining coping. This can lead the coping literature in the gig-work context to face the same problems as those existing in mature contexts (i.e., lack of consensus). For instance, the coping literature in the academic context has shown that the concept of coping is fragmented due to relying on lists of multidimensional lower-order categories (e.g., ways of coping or coping strategies) that likely serve many functions without making efforts to explain how they are functionally related or different. This approach has made it difficult to scrutinize, aggregate, or compare this knowledge and will lead to a fragmented view of coping [16]. In a series of studies conducted in the context of academic work, Skinner and their colleagues [16,17,18] overcome this problem by suggesting that hierarchical structures of coping help connect lower-order categories to higher-order categories such that adaptive processes should be the focus of research. Borrowing from their suggested hierarchical structure of coping based on the motivational action theory of coping [16], we suggest a radar framework of coping in the context of gig work that would fit the current literature and has the potential to extend future research, both theoretically and methodologically. Relying on self-determination theory (SDT) [3,19,20,21,22] and the motivational action theory of coping [16], we suggest a radar structure for gig-worker coping that explains how gig work impacts SDT’s basic psychological needs for autonomy, competence, and relatedness, and how gig workers react to them as “adaptational problems,” to protect their well-being. We highlight those reactions or coping families that may decrease harm and increase well-being and those that may exacerbate harm.

Our aim is to empower future research with a categorization process of coping based on empirically tested theories of behavior to avoid conceptual fragmentation of coping and produce knowledge that can be compared and integrated. Our radar framework extends research with a framework for data analysis, particularly for examining online forum comments. Propositions developed in this paper can be tested using validated measures of the constructs (see [16]).

## 2. What Is Coping?

One of the most studied concepts in almost all situations concerns “how people cope with stressful situations”. The study of coping is fundamental because researchers agree that coping can reduce or amplify the effects of negative events and conditions on emotional distress and well-being [16]. As Skinner, Edge, Altman, and Sherwood’s [16] comprehensive review of systems of coping shows, the fundamental problem in the study of coping is that it is not a specific behavior that can be explicitly observed; instead, it is an organizational construct incorporating numerous actions that individuals take to deal with stressful experiences. Scholars are usually interested in conceptualizing or measuring “ways of coping,” which are categories based on selected dimensions of coping which classify the ways people cope [16]. The lack of consensus on dimensions of identified ways of coping is one of the main issues in the literature that hinders the progress of our understanding by making it impossible to compare results from different studies or aggregate them [16]. To overcome these and multiple other problems about lack of consensus about coping dimensions, categories, and mechanisms, Skinner et al. developed and suggested a comprehensive and exhaustive structure of coping in which categories are functionally homogenous (i.e., all ways of coping within a category serve the same set of functions) and distinct (i.e., categories are different based on the set of functions they serve). 

The motivational conceptualization of coping formulated by Skinner et al. [18] defines coping as action regulation in psychological stress, where action consists of behavior, emotion, and orientation. In other words, coping refers to the ways people “mobilize, manage, energize, guide, channel, and direct their behavior, emotion, and orientation in stressful circumstances, or how they fail to do so”. (p. 401). This theory assumes that coping is a “strategy of adaptation” [1,16] to the social world and involves making compromises between total triumph over the environment and total surrender to it. By coping, individuals serve larger evolutionary functions, such as securing adequate information about the environment or escaping from a potentially dangerous transaction [16].

Through multiple deductive and inductive studies (starting from the academic context of children), Skinner and their colleagues [16,17,18] introduced a coherent set of categories of coping that connects the lowest level of analysis or “instances” of coping to the highest level or adaptive processes that intervene between stress and its psychological, social, and physiological outcomes [16]. In other words, the hierarchical structure of coping connects the countless changing real-time responses that individuals use in dealing with specific stressful transactions, such as “I read everything I could find about it,” to strategies of adaptation, such as “continuing to secure adequate information about the environment”. Before we delve into more details in describing this structure, we next introduce the gig work context and, finally, adapt this structure to the gig work context to see whether the same structure can be concluded from gig workers’ “ways of coping” or “coping strategies”.

## 3. Gig Economy, Gig–Worker Experience, and Coping

Gig work involves at least three stakeholders, including the platform, clients, or “buyers”; independent workers or “suppliers”; and the digital labor platform organization, which serves as an intermediary coordinating buyers and suppliers [3]. Platforms enable clients to receive local and virtual (global) services from low- and high-skilled workers. For instance, “Uber” and “Lyft,” which provide local transportation and delivery services, mostly involve low-skilled workers. At the same time, platforms such as “Upwork” and “Fiverr” enable individuals and organizations to outsource high-skill tasks—such as graphic design or data analysis—to the anonymous global workforce [3,9,23]. In these platforms, algorithms based on programmatic processes play a key role in matching workers with potential clients and evaluating and managing the transparency of the skill and performance of workers (e.g., ratings and reviews) [23,24]. While digital platform organizations advertise the merits of gig work as self-employment, entrepreneurship, and financial independence [23], scholars critique these new work relationships, stating that there is a high incongruence between the rhetoric of entrepreneurial freedom and the lived experience of gig workers that mostly includes highly controlled, precarious, and low-paid work [9,25]. Research on the working status of gig workers shows that the lived experience of their work identifies them not as entrepreneurs or self-employed but as bogus or false self-employed workers. False self-employment “is where workers are self-employed but have a de facto employment relationship, economic dependence (where a worker generates their income from one or mainly from one employer), and personal dependence (i.e., subordination and lack of authority on working methods, the content of work, time and place)”. [26] (p. 2) The OECD [27] (2000: 156) describes false self-employment as an employment method to declare workers as self-employed to reduce tax liabilities or employers’ responsibilities. The categorization of some gig workers as false self-employed explains some coping behaviors by them that are unlikely to happen for self-employed workers. For instance, while collective action is unlikely for self-employed workers due to a lack of structured antagonism, protests by Uber, Deliveroo, and Foodora workers have been started due to the de facto employment relationship that these workers experience [10].

Kellogg, Valentine, and Christin’s [24] influential review of algorithmic control shows that algorithmic control in general, and platforms in particular, control workers through six main mechanisms called 6R, and “they are using algorithms to direct workers by restricting [(e.g., Uber narrowing shift, ride, or delivery choices)] and recommending [(e.g., Uber’s individualized and real-time nudging to actively make drivers go home)], evaluate workers by recording [(e.g., Upwork’s use of real-time metrics to monitor workers,)] and rating [(e.g., User-generated rating systems of most labor markets)], and discipline workers by replacing [(e.g., Uber instantly penalizing drivers for rejecting orders, Upwork closing accounts of workers regularly submitting work proposals but not winning projects)] and rewarding [(e.g., Uber’s Opaque and manipulative rewarding system)]. Algorithmic control and platform diminish worker well-being due to the ambiguity, anxiety, and insecurity resulting from the lack of a set of stable work arrangements, limited worker protections, and little to no pathways to promotion” [14,15].

### Coping with Algorithmic Control

The gig work literature has shown that gig workers are not passive regarding need frustration, and they respond in a variety of behaviors [5,9,28,29]. For instance, gig workers investigate algorithmic systems, creatively game the system, work around constraints, and mobilize the algorithmic platform to gain autonomy [13]. In their study of gig workers on the Upwork platform, Bucher et al. [23] found that the algorithmic management’s highly complex and non-transparent features lead workers to engage in anticipatory compliance practices. In doing this, workers take two different approaches toward pacifying the algorithm. In the first approach, workers use direct compliance practices, which involve direct engagement with the algorithm, implying no significant action toward clients. Staying under the radar and purposely curtailing client outreach are other compliance practices using a direct approach. In the second approach, workers use indirect compliance practices, seeking to receive clients’ prompt feedback, which in turn impacts the workers’ rating from the algorithm. Undervaluing work and keeping emotions in check are the indirect compliance practices of gig workers [23].

In another study on coping with algorithms, Jarrahi and Sutherland [13] found that the information asymmetries in digital platform work make it difficult for gig workers to understand how algorithms make important decisions, such as work evaluation and assignment. To understand how to interact effectively with algorithms, gig workers engage themselves in three types of activities, sense-making, circumventing, and manipulating algorithms [13]. Muszynski et al. [30] have studied the impact of COVID–19 on Polish gig workers’ life and work experiences. They have shown that based on access to resources, access to institutional capabilities, and orientations, gig workers take different strategies in dealing with substantial fluctuations of demand on digital work platforms. The three coping strategies discussed in their article are (1) staying loyal and not challenging the perils of the platform; (2) hybrid strategies: staying loyal and voicing complaints; and (3) exiting [30].

Pregenzer and their colleagues [31] have studied ride-hailing drivers and their reactions to algorithmic control. They have used two antecedents of worker reactions, compatibility and coherence, as proposed by Gill [32]. Compatibility is the extent to which the workers perceive harmony between their interests and values and algorithmic control, and it will be either fulfilling or suffering. Coherence is the extent to which workers experience controlling mechanisms as fragmented (pulling in different directions towards contradicting goals) or unified (unison toward a consistent goal). Based on the coherence and compatibility of control mechanisms, four ideal–typical worker reactions have been proposed in a two-dimensional matrix: (1) blending, to embrace controls and exert efforts to satisfy customers, (2) bridging, to try to reduce incoherence by closing the gaps, (3) distancing, to resist moderately and avoid certain aspects of control, and (4) separating, to voice allegations and resentment, or quit the job [31,32]. 

The six studies of coping that we reviewed in the previous paragraphs resulted in fourteen coping strategies, so it is likely that relying on lower-order categorizations for coping will make it difficult to aggregate the literature. We used data (findings and interpretations) on gig-worker coping strategies to investigate whether gig-worker coping strategies can be categorized into the hierarchical structure of coping proposed by Skinner and their colleagues [16,18] in the academic context.

## 4. The Hierarchical Structure of Coping

### 4.1. Basic Psychological Needs

Self-determination theory (SDT) argues that humans have the potential for growth, integration, and well-being while also being vulnerable to defensiveness, aggression, and ill-being [20]. Within SDT, the nutriments for growth are specified using the concept of basic psychological need satisfaction [21]. The set of these basic needs is very limited because any basic need candidate or specific desire should meet all of nine strict criteria (e.g., “felt need satisfaction and need frustration should predict the thriving and ill-being of all individuals, regardless of differences in socio-demographics, personality, cultural background or need strength”; see [20] (p. 4) for full reference) to be assigned the more formal status of a basic psychological need [22]. In a brief sense, regarding basic psychological needs, “satisfaction is not only conducive to, but essential for individuals’ well-being, while its frustration increases the risk for passivity, ill-being, and defensiveness”. [19,20,22] (p. 1).

The current list of these needs consists of the need for autonomy or experiencing volition and willingness; relatedness or experiencing warmth, bonding, and care from significant others; and competence or experiencing mastery and ability to cause desired outcomes [22,33,34,35,36]. The satisfaction of basic psychological needs represents individuals’ psychological, energetic resources, and as such, stimulates individuals’ well-being and performance, whereas thwarting those needs has an energy-depleting effect, leading to lower performance and well-being [21,37,38]. This premise has been tested by various studies in different cultures and life domains and across different occupations, providing support for the relationship between basic needs and burnout [21,39,40,41].

The hierarchical structure of coping is based on a motivational action theory of coping that argues that three classes of concerns related to individuals’ basic psychological needs (i.e., autonomy, competence, and relatedness) trigger organized biobehavioral responses or “families of coping” [16,17,18]. In line with SDT, we can categorize adaptive processes as efforts to adapt to the loss of each need as adaptive processes that (a) coordinate actions with the contingencies in the environment (concerned competence), (b) coordinate reliance on others with the social resources in the environment (concerned relatedness), and (c) coordinate preferences with the options available (concerned autonomy) in the environment.

Rockmann and Ballinger [42] tested the main premise of SDT for the highly skilled, on-demand work context, and found that to the extent that on-demand work fulfills one’s basic psychological needs, workers will develop intrinsic motivation [3,5]. Jabagi et al.’s [3] nuanced literature review on how basic psychological needs are facilitated or impaired in platform work included a wide range of platforms and integrated the job characteristics model and enterprise social media research in relation to SDT to suggest that platform organizations can motivate workers by the thoughtful design of their digital labor platforms and the integration of social media technology.

### 4.2. Adaptational Processes

Species face multiple fundamental adaptational problems that need to be addressed for their survival [1,16], and internal or external threats to well-being are some of those issues [1]. Coping, as one adaptive solution, includes “a complex psychobiological reaction that fuses intelligence with motivational patterns, action impulses, and physiological changes that signify to both the actor and observer that something of significance for well-being is at stake in the encounter with the environment” [1] (p. 615). A person–environment relationship involving a particular kind of harm or benefit generates action tendencies relevant to the specific conditions of harm or benefit and stimulates (motivates) the organism to behave in ways that enhance its potential to survive and flourish. 

### 4.3. Families of Coping

The hierarchical structure consists of two intermediate levels. Lower-order categories or “ways of coping” are categories that classify “instances of coping” (items or observations) into mutually exclusive categories (e.g., problem solving, rumination). Higher-order categories are “families of coping” that classify ways of coping according to their adaptive functions. Each family of coping is organized around (1) whether the concern is appraised as a challenge versus a threat and (2) whether the ways of coping are targeted at oneself or one’s context. The criteria for family membership are behaviors, emotions, and orientations that each pattern of appraisal triggers. For instance, an appraisal of a “challenge for autonomy” will be accompanied by an “Accommodation” or a “Negotiation” coping family. Still, if the level of stress related to the concern increases such that it becomes appraised as a “threat to autonomy,” it will be accompanied by “submission” and “opposition,” depending on whether a person decides to target the self or the context. The result is higher-order families of coping organized around three basic psychological needs, two appraisals of challenge vs. threat [43], and two coping targets of self and context. These families of coping will be illustrated in detail in the next section, through our radar representation of this hierarchical structure.

## 5. Toward a Radar Structure of Gig-Worker Coping

**Figure 1:**
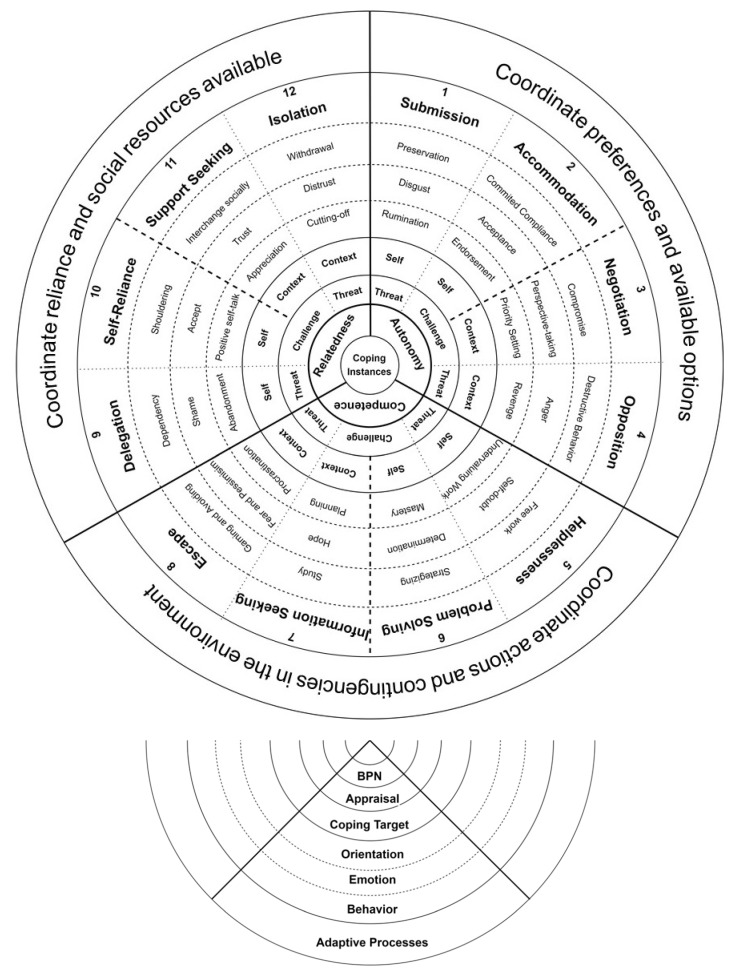
A radar view of 12 families of coping in gig work and a corresponding reading guide based on multiple dimensions of coping involved. Note: BPN = basic psychological needs.

Borrowing from Skinner et al. [16,17,18] the academic context of gig work, we argue that the hierarchical structure of coping suggested by the authors can be a useful theoretical lens through which to study coping. Following Whetten, Felin, and King [44], in the practice of theory borrowing, we maintain the theory’s nature. Still, the uniqueness of gig work compared to the academic context that can affect the theory’s logical structure will be investigated for all dimensions of Skinner et al.’s [16] hierarchical structure of coping. Figure 1 displays our suggested structure for the study of coping adapted from Skinner and their colleagues [16]. To make it easier to understand and include more examples, we have modified it to a radar format. Surprisingly, the original framework, while very comprehensive and informational, has not been used by other researchers, and one reason may be the complexity of the table that makes it hard to understand, especially for researchers outside the psychology field. 

We believe a radar arrangement of the dimensions of coping makes it easier to understand, remember, and interpret. Our proposed radar of copings in gig work includes 12 families of coping identified based on (a) three basic psychological needs, (b) two appraisals of challenges or threats, and (c) two targets, self or context. These 12 families of coping connect first-order instances of coping to higher-order adaptive processes of coping, and each involves a set of related behaviors, emotions, and orientation, and they are arranged in three sets of four. The radar is designed to highlight the critical roles of three basic psychological needs and constructive coping families, those that are triggered as “challenge” appraisals of contextual factors. These coping families are highlighted in our framework. In the radar, the first set involves concerns for autonomy and is triggered when workers feel pressured to act against their will. The second set of coping families involves concerns about competence and is triggered when workers perceive a lack of information to predict work outcomes in the context. The third set of four involves challenges and threats to relatedness and is triggered when workers feel that they are not trusted or cannot trust important others.

### 5.1. Basic Psychological Needs: Autonomy, Competence, and Relatedness in Gigs

According to self-determination theory, contexts that frustrate the needs for autonomy, competence, and relatedness will lead to high levels of psychological stress and consequently to decreased motivation and well-being [16,18,19,22], (p. 1). Table 1 compares contextual factors that harm children’s basic psychological needs at school [18] with those of the gig-work context (with corresponding references). Skinner and Wellborn [18] reviewed the literature on the context that threatened the three basic psychological needs and suggested that students’ need for autonomy gets depleted in interactions where children are pressured, for example, by rules or rewards, to behave in certain ways or to express certain feelings. Social contexts in which information is lacking about how to produce desired effects (e.g., when the rationales for rules are not explained, when children are asked to attempt too difficult activities, when strategies for producing outcomes are not well-specified, when opportunities for independent attempts are not sufficient, or when feedback for strategy implementation are not provided) deplete the need for competence. Need for relatedness can be depleted in multiple situations; for instance, when teachers ignore or overlook or fail to communicate their regard and affection for children, when the people at school or its general climate are cold, distant, and uncaring, or even hostile and rejecting of children or when parents express no interest in their children’s schoolwork or experiences.

Similarly, we can see from the gig worker literature that rewards and incentives to motivate them to show specific behaviors (accept more tasks and create specific results) can undermine autonomy. In the same way, lack of feedback and lack of information about how to perform the task undermines the need for competence, and finally, a cold working climate for gig workers (e.g., they have no coworkers, the platform does not care about their affect and well-being, and they are distant from their family because of overwork) undermines the need for relatedness. Some need-depleting factors are identified in the gig literature that does not relate to the academic context. In the work context, an important controlling factor is compensation and payments. Platforms undermine autonomy through controls on pay rates and the financial dependency of workers on the platform to motivate them to work more.

**Contexts that threaten the need for “autonomy**” for children and adults are characterized as those that pressure them with rules or rewards to enact specific behaviors, express certain feelings, or accept certain goals or courses of action [16,18]. Jabagi et al. [3] argue that an algorithmic architecture can support and impede basic psychological needs based on its design. They argue that designs that motivate the worker to maintain a high gig acceptance rate can have a negative effect on worker autonomy. In regard to the need for autonomy, workers need to perceive that they are the ones who decide what tasks to choose or reject; and when, how, and how much to work [3]. When an algorithm penalizes workers for acceptance or cancelation rates, or when it motivates them with rewards to do so, they will feel pressured to take actions against their will by algorithmic control [3,9,24], and this control will deplete their need for autonomy. Scholars identified another controlling aspect of gig work related to low pay and involuntary participation in gig work. Workers who are financially dependent on algorithmic work will perceive their work context as highly controlling [3,9,24], leading to frustration of the need for autonomy. 

**Contexts that threaten the need for “competence”** for children and adults are characterized as those that lack the information about producing desired effects and are inconsistent, random, discriminatory, or unfair [16,18]. In the context of platform work, there is no human as a supervisor or mentor to give constructive feedback to the workers. Algorithm-generated feedback is not usually constructive due to the differences in perceived functional significance between workers and platform organizations. Most digital labor platforms do not have a rating system that can support workers’ need for competence [3]. Workers may receive positive feedback from a client; however, there is no guarantee.

**Contexts that threaten the need for “relatedness”** for children and adults are characterized as those in which they do not receive enough care, respect, communication, and affection from their teachers, classmates, or parents [16,18]. Platform workers do not have the opportunity to feel like insiders in the platform organization. They often experience a sense of isolation due to their work’s “remote” nature, which leads to workers’ burnout and frustration. Although the workers’ need for relatedness can be fulfilled through their interactions with clients, this is not the case for all platforms [3,12]. Zhang et al. [12] found that the drivers feel that their well-being support is unaddressed in their study of Uber drivers. Platform organizations using algorithmic capabilities to address and support drivers’ well-being were identified as some of the key resources lacking in gig work.

### 5.2. Appraisals of Challenge Versus Threat

The gig-worker coping literature uses the terms “challenge” and “threat” interchangeably, whereas the established literature of academic and traditional work context suggests that risk factors, demands, or negative experiences differ based on whether they are perceived as challenges or threats [16,43,45,46]. From an SDT perspective, challenges are known stressful situations that can initiate the motivational process and support health [45] through ways of coping that promote constructive engagement with stressors (e.g., planning, negotiation, and sense-making [16,18]). Threats (or hindrances in the job demands-resources model terminology), on the other hand, impair health and predict burnout directly or through emotionally extreme coping mechanisms that can be risk factors for health [16,46]. 

These studies suggest that appraisals of challenge versus threat can affect well-being and health either directly by frustrating basic psychological needs or by triggering ways of coping that deal harshly with the self (e.g., self-blame and social isolation) or with a stressful situation (e.g., blaming others and negative thinking) that are instrumental for managing stress and negative emotions, but they are detrimental for health over time. The first group of ways of coping contributes to well-being through the construction of coping resources, such as self-reliance, confidence, and interpersonal trust. In contrast, the prolonged use of the second group of ways of coping, over time, contributes to the accumulation of vulnerabilities, such as low self-efficacy or interpersonal hostility [18]. For instance, if gig workers perceive a stressful transaction with a client or algorithm as a threat rather than a challenge to increase competence, problem-solving coping will give way to helplessness; with increased pressure, negotiation will become aggression, and with an increased feeling of loneliness, support seeking will give way to the isolation coping.

We argue that it is important to differentiate coping strategies that are initiated based on perceptions of challenges from threats in gig work, since it would help researchers to identify those characteristics of an algorithm that should be changed or intervened, as they can only usefully be perceived as threats when considering those they affect, and with thoughtful design of the algorithm, challenging aspects of the job may even elicit engagement and well-being. Negative feedback, time pressure, and cognitive demands have good potential to be perceived as challenges, whereas role ambiguity, job insecurity, and interpersonal issues are more likely to be perceived as threats by workers [45]. For instance, scholars have reported contradictory results regarding gamification in gig work. While some scholars argue that gamification strategies used by platforms to make drivers more efficient and work harder increase their extrinsic motivation (i.e., to play for financial gains [47]) or even can increase intrinsic motivation in a specific type of gamification, namely, “badging” [14], others argue that gamification makes drivers feel like they work in an unequal system, a system with unclear rewards and objectives which may deplete their sense of autonomy and competence [12]. These contradictory results show the importance of gamification strategies being designed to support well-being but also suggest the possibility that the designs of games, based on their difficulty and controlling nature, can be perceived either as challenging or threatening by workers.

### 5.3. Targets of Coping: Self and Context

Most typologies of coping involve distinguishing coping based on whether the coping targets the self or the context. For example, within appraised challenges to autonomy, one can target the self by attempting to accept and accommodate control (e.g., keeping emotion in check, [23]; Blending, [31]), or one can target the context by negotiating with clients for higher ratings or compromising their emotions in their conflicts with clients [23]. For example, when individuals appraise a challenge to “competence,” they can target the self by attempting to leverage resources to produce desired outcomes (i.e., problem solving), or one can target the context by trying to make sense of available resources (i.e., information seeking).

### 5.4. Adaptive Functions of Coping Families

The glue binding families of coping and adaptive processes is a widely held assumption on most views of coping: “that the repertoire of human responses to stress has been shaped by evolution”. [16] (p. 244). The final step is to explain the functions that each family of coping serves in its evolutionary role. Skinner and their colleagues [16,18] organized the functions of the 12 coping families into three functions that help an organism adapt to its environment under stress. In SDT, adaptive processes are categorized as efforts to adapt to the loss of each need as adaptive processes that (a) coordinate actions with the contingencies in the environment (concerning competence), (b) coordinate reliance on others with the social resources in the environment (concerning relatedness), and (c) coordinate preferences with the options available in the environment (concerning autonomy).

## 6. Does the Radar Structure Fit the Current Data? 

We searched for gig-worker coping literature to see how authors conceptualize coping strategies in response to the frustration of each basic psychological need and what dimensions were identified. For the purpose of this study, we selected a handful of studies that had enough explanations and participant quotations to help us identify the “emotions, orientations, and behaviors” of strategies and classify them based on Skinner’s [16] hierarchical structure of coping. We coded the data and interpretation of selected papers based on the described emotions, orientations, and behaviors. Using the radar structure and the appraisal pattern suggested by Skinner and Wellborn [18] (Figure 2, p. 402), we clustered these groups into families of coping.

An overview of the selected papers, their coping strategies, and the corresponding “coping families” based on our interpretation is presented in Table 2. As shown in the table, coping with relatedness frustration has been the least studied in the literature. Our search of the main databases of literature did not result in finding coping strategies that tap into our descriptions of “delegation” and “isolation,” and this is problematic, as the gig-work context has been shown to be extremely exhausting in terms of the need for relatedness [3,25], and workers’ means of coping with isolation matter to developing strategies of well-being support. Our analysis shows that researchers have reported important coping strategies in the field, and a hierarchical structure of coping and motivational action theory of coping have the potential to integrate and extend the current coping literature.

### 6.1. Submission: From Threatened Autonomy to Self-Targeted Coping

The first set of four on our radar relates to the need for autonomy. When a challenge to autonomy is appraised, individuals either “accommodate” (self-targeted coping) or “negotiate” for their autonomy. When the source of stress is appraised as a threat to autonomy, two rigid families of coping may arise based on the target of coping. “Submission” is a more rigid form of “accommodation” when one gets obsessed with negative thoughts related to loss of autonomy, and “opposition” is a more rigid form of “negotiation,” where “revenge, anger, and aggression” help the individual with extreme loss of autonomy.

As a higher-order family of coping, “submission” refers to pressuring the self to respond in overregulated ways; thus, it is a pattern of emotion regulation of “self-blame or disgust” and regulates “rumination and obsession” and behaviors such as “rigidity”. Rumination refers to a passive and repetitive focus on the negative and damaging features of a stressful context [16]. “I don’t know what I want” is a sample pattern of appraisal for submission. When individuals perceive high levels of frustration with their autonomy, they engage in draining and constant ruminative and negative thoughts and pressure themselves with self-blame emotions and become obsessed with the tasks they are doing. 

In the context of gig work, Walker et al. [25] described a group of gig workers who seem to show “involuntary engagement” as a stress reaction represented by the username “mulder99,” who is critical of the company and drives for multiple rideshare companies, and is very active in the forum and embodies the values that “the best thing drivers can do to better their situation is to simply work longer hours” [25] (p. 11). This framework suggests that it is important to differentiate “submission,” which is involuntary engagement with gig work, from a voluntary and constructive form of engagement (e.g., accommodation) which is triggered when the demand is perceived as a challenge rather than a threat. 

### 6.2. Accommodation: From Challenged Autonomy to Self-Targeted Coping

As a higher-order family of coping, “accommodation” refers to adjusting one’s preferences to current constraints; thus, it is a pattern of emotional regulation of “acceptance,” an orientation regulation of “commitment or endorsement,” and behaviors such as “committed compliance or cooperation”. “I will decide” is a sample pattern of appraisal for “accommodation” and contributes to an endorsement of control and showing flexibility in behavior. These individuals emotionally “accept” the context and reflect and rely on its benefits and responsibilities.

Compliance is a prevalent theme in gig work literature, but it is important to differentiate different forms of compliance based on whether it is oriented toward endorsement and commitment (e.g., committed compliance) or self-doubt and staying away from the threat (e.g., helplessness). Bucher et al. [23] showed that to ensure their continuous and successful participation in the platform, workers adjust their behavior to comply with the expectations of clients. For instance, in their study of digital freelancers on Upwork and Fiverr, Bucher et al. [23] showed that when facing clients’ unfair treatment or negative reviews, some workers decide to swallow their negative emotions to maintain their reputation on the platform. This form of accommodation has been called “keeping emotions in check” (p. 13). Pregenzer et al. [31] described a group of workers “blending” in with the algorithm that have criticisms of the ride-hailing business and would quit if they could find another job, but since finding another job is difficult, they stay and play under the rules.

### 6.3. Negotiation: From Challenged Autonomy to Context–Targeted Coping

Families of coping that promote constructive engagement with stressors and are triggered in response to an appraised challenge to autonomy are “Accommodation” and “Negation”; the former concerns targeting the self to “accept” the situation and endorses its benefits, and the latter concerns targeting context by taking to account other’s perspective, setting priorities based on available options and making compromises according to the priorities. As a higher-order family of coping, “negotiation” involves actions such as proposing a compromise, bargaining, and persuasion. Individuals, through these strategies, deal with the context to generate more options for themselves. “I will reduce the pressure” is a sample pattern of appraisal for this category.

In the context of gig work, Bucher et al. [23] found that workers negotiate directly with clients and ask for positive (or, in case of negative interactions, not negative) feedback by “pointing out how crucial their feedback is or how much a worker relies on a good feedback” (p. 14). For such negotiations, they “compromise” their feelings and prioritize high ratings and staying on the platform. Muszyński and their colleagues [30] found that some highly skilled platform workers engage in negotiations with clients to mitigate the stress of gig work, and, more specifically, the stress of lack of protection. They arrange their relationships with clients to recreate conditions for a more stable flow of income or “quasi-standard employment relationship”. For instance, Matylda, a gig researcher, voiced her problems to the clients concerned to make them plan their commissions in advance or convince them to pay her for previously unpaid research work.

### 6.4. Opposition: From Threatened Autonomy to Context–Targeted Coping

When individuals appraise the context as a threat to their autonomy, and they target the context for coping (e.g., “The world is hostile”), a set of action regulations focusing on orientation of “reactance or revenge,” emotions of “anger or blaming others,” and “aggressive behaviors” will be triggered. 

In the context of gig work, workers show high-intensity resistance to remove themselves from control and are unable or unwilling to find mitigating tactics to engage in the “opposition” family of coping, which includes behaviors such as voicing allegations and resentment, quitting, or destructive behavior accompanied by distrust and disgust towards the platforms—and sometimes the users [31]. Such aggressive and destructive behavior can be seen in quotes from a driver in their study: “At any time I suspect an impending 1 star, I cancel for a random reason like my cars check low oil pressure light is flickering on, etc. Sounds like you swiped right and started it prematurely, I would’ve said GTFO, timed out and collected my precious cancel fee, don’t play college kid games” [31] (p. 11).

### 6.5. Helplessness: From Threatened Competence to Self–Targeted Coping

When workers appraise a “threat” to competence, they will trigger extreme forms of regulation, such as the emotion of “self-doubt” relative to “challenge” appraisals that trigger constructive coping regulations, such as the emotion of “determination”. “As a higher-order family of coping, helplessness refers to a set of actions organized around relinquishment of control” [16] (p. 27). “I cannot accomplish that” is a sample pattern of appraisal for “Helplessness”. If threatened, competence triggers self-targeted regulations, a pattern of behaviors (e.g., random attempts), emotions (e.g., self-doubt), and orientations (e.g., Confusion) that corresponds to relinquishment of control. These workers perceive the demands of the work context are beyond their capabilities to have hope and engage in constructive behaviors; they are confused (orientation), and the strategy they choose to regain their well-being is to “give up control”. As their appraisal of context conveys to them the “fact” that they are not competent in their work, they develop emotions of self-doubt. In academic literature, behaviors associated with self-doubt and confusion are identified as “inaction or passivity” [16]. 

In the context of gig work, Walker et al. [25], in their study of Uber drivers in Australia, found that Uber Australia’s algorithmic control and rating system involves penalizing for negative feedback and low ratings, creating a sense of hopelessness among workers. Drivers who believe that there will be no possible way to seek improvement in work conditions through collective action lose hope and become disheartened. This state of hopelessness leads drivers to give up on their efforts to improve their work conditions and refill their depleted need for competence [25]. Bucher et al. [23] identified a group of gig workers who cope with threats of the algorithmic rating by undervaluing their work and providing free work in the form of billing fewer hours than the actual amount of time or carrying out fixes for free that often take substantial amounts of time. These workers feel that they must do everything possible to avoid a negative rating and would “rather fix [problems] for free than charge the client” [23] (p. 13) to improve the client experience and acquire a favorable rating. 

### 6.6. Problem Solving: From Challenged Competence to Self–Targeted Coping

In the case of challenged competence, if workers target the self and engage in individualized actions to respond to psychological stress, they will use the “problem solving” family of coping. The orientation is to gain “mastery” in work with active attempts to produce effects and determination. “I will accomplish that” is an example of an appraisal pattern associated with this family of coping that includes “problem-focused” ways of coping discussed by almost all categorizations of coping [16]. 

In the context of gig work, researchers report high levels of information asymmetry and opaqueness in ways to earn higher ratings [3,13,24]. Jarrahi and Sutherland [13] showed that gig workers use different problem-solving strategies to fill the inefficiencies of algorithms and make better use of their functionality or even make alternative uses of certain features. For instance, while workers often think that Upwork’s proficiency tests (e.g., knowledge of English Grammar or user experience design) are not accurate manifestations of their skills and avoid taking them, one participant found that taking the tests made him more outstanding in search results for terms related to his skill.

### 6.7. Information Seeking: From Challenged Competence to Context–Targeted Coping

While workers appraised “threats” to competence trigger rigid families of coping such as “helplessness,” a “challenge” appraisal of sources of psychological stress triggers constructive coping families. In the case of challenged competence, they are “information seeking” and “problem solving”. As a common way of coping in response to disease, information seeking, as a family of coping, “refers to attempts to learn more about a stressful situation or condition, including its course, causes, consequences, and meanings and strategies for intervention and remediation”. [16] (p. 27). “I will learn” is a sample pattern of appraisal for “information seeking”. If challenged competence triggers a context-targeted coping family, “information seeking” regulations, a pattern of behaviors (e.g., study), emotions (e.g., hope), and orientations (e.g., planning) will be exercised to reduce stress in the short run and regain well-being in the long run by trying to reduce the information asymmetry between individual and context.

In the context of gig work, Jarrahi and Sutherland [13] showed that workers put in significant amounts of time and effort to understand the platform’s various ratings, timeframes, policies, and procedures. The “sense-making” way of coping in Jarrahi and Sutherland’s [13] study involves behaviors such as “attempt[ing] to get a better understanding of algorithms by approaching platform from the client-side of the platform” (p. 6) or “seeking advice about how to deal with difficult clients or Upwork [platform] policies” (p. 7) on social channels such as online forums. This family of coping has been found to be very important in the context of gig work since it sets the stage for other ways of coping with the algorithm. For instance, Jarrahi and Sutherland [13] argue that sense-making strategies open the black boxes of algorithms to effectively work with and around them. In other words, it is after studying the algorithm and getting familiar with the system and the loopholes that workers decide based on their resources, capabilities, and beliefs to either “comply” with the algorithm or “escape”.

### 6.8. Escape: From Threatened Competence to Context–Targeted Coping

When workers appraise a “threat” to competence and target the context for coping, they will act based on another rigid form of coping, namely, “escape,” which “includes efforts to disengage or stay away from the stressful transaction” [16] (p. 27). “The world is unfair” is a sample pattern of appraisal for “escape” and includes “pessimism and fear” and avoidant actions. 

In the context of gig work, Bucher et al. [23] found that some workers purposely avoid some tasks and clients in response to a sense of paranoia that leaves workers wanting to over-adjust their behavior to be on the “safe side” and not trigger any algorithmic scrutiny. One user even stated that algorithmic functions “are just WIZARDS at recognizing and punishing” [23] (p. 12). The workers forgo potential income to not receive “bad feedback, or almost worse, no feedback at all” (p. 13). For instance, workers inspect clients to recognize and avoid potentially risky clients (e.g., those with a history of negative feedback). As explained earlier, for the “information seeking” coping family, an important strategy is studying clients and getting advice from other workers in the online community to help them to recognize potential “red flags” in client profiles early on [23]. Labeled as a “staying under the radar” way of coping, these strategies are two-fold: the fear of incurring one bad rating and/or the fear of suspension or ban from the platform. Indeed, when a worker’s score is fragile, one more bad rating would lead to being banned from the platform. Pregenzer et al. [31] referred to the combination of client feedback through ratings and reports, with account deactivation as one of the control bundles that frequently causes frustration. 

### 6.9. Delegation: From Threatened Relatedness to Self–Targeted Coping

The next set of four families of coping is centered around the psychological need for relatedness or the need to “love and be loved”. When the context is perceived as a threat to relatedness and the individual targets the self to cope (e.g., “I am alone”), a set of regulations will be triggered to cling to and be dependent on someone close to them. As a higher-order family of coping, “delegation” refers to attempts to get someone else to do the work [18] centered around emotional regulation of “self-pity or shame” and the tendency to “abandon” stressful situations. It is important to note that the “Delegation” coping family is differentiated from “support seeking,” as the former is a more rigid form of behavior, and the individual tries to be extremely dependent on others, rather than seeking comfort or emotional support to reduce the feeling of neglect [16,18]. As explained earlier, this category has only been identified among children (e.g., being dependent on parents to do homework), and it is an open question whether gig workers delegate some or all parts of their work. For instance, is it possible for a food delivery driver that had just an accident with his car to call his friend to come, take food, and deliver it instead of him to avoid a negative rating for cold food? We believe if they perceive that neither the platform’s company nor the food company “cares” about their problem and the accident, they will do so. In the case of highly skilled workers, a worker can also delegate parts of their job based on the level of monitoring imposed by the platform. Based on these speculations, we decided to keep “delegation” on our “radar” and suggest future research for further investigation of this phenomenon.

### 6.10. Self–Reliance: From Challenged Relatedness to Self–Targeted Coping

In the case of a challenged relatedness, individuals can target the self rather than context and turn to themselves (e.g., positive self-talk) or those they love to receive emotional support [16]. In the context of gig work, nonwork relationships, such as families and friends, have been found to be extremely important to cope with the challenges of work, providing workers with a variety of resources, such as psychological and physical resources, and social capital [7,48]. For instance, having access to financial resources like a spouse’s job can reduce the pressure felt by the gig workers, or having emotional support from the network of friends and family can appease the stress felt by workers facing their work demands [7,49].

### 6.11. Support Seeking: From Challenged Relatedness to Context–Targeted Coping

In the case of appraised “challenge” to relatedness, the coping families triggered are constructive, and unlike “delegation” or its opposite rigid form of regulation, “isolation,” the regulations involve optimistic emotions, such as acceptance and trust. The (social) support seeking family of coping has been studied under labels such as social support, proximity seeking, or help seeking [15] and involves actions and emotions to actively seek support the individual in the context (targeting context) and to trust others. “I will connect/love” is an example of an appraisal for this family of coping and involves emotional regulation based on “trust” and an orientation toward an appreciation of others. 

In the context of gig work, the work being performed separately from coworkers, compared to traditional forms of work, makes it challenging to enable feelings of stable personal connection and belonging [3,7,50]. Ashford and their colleagues [6] argue that for workers to thrive in the digital economy, independent workers must construct relationships agentically, including other independent workers with similar skills, potential clients, supporters, and employers. This network of support provides human connection and allows workers to buffer the stress of work [50] and supports people to cope with the novel challenges they face, and offers them a sense of connection [7,51]. In the case of Uber drivers, opportunities for social support and sharing experiences, news, and concerns on the job can be found on the roads, in cafes, at waiting points, and, most importantly, in social media forums and chatrooms [25].

### 6.12. Isolation: From Threatened Relatedness to Context–Targeted Coping

When a context is perceived as a threat to relatedness and the individual targets the context to cope (e.g., “The world is cold”), a set of regulations will be triggered to isolate the self from the context that is the source of threat, and there is no hope for any support from it. As a higher-order family of coping, “isolation” refers to attempts “aimed at staying away from others or preventing other people from knowing about a stressful situation or its emotional effects” [16] (p. 27).

In the context of gig work, we did not find studies reporting “isolation” as a coping strategy in the sense that these strategies “isolate workers from their context in response to lack of care from the context”. While isolation is a prevalent theme in the studies of gig work, they mainly refer to the physical separation of workers due to the nature of their job, or Uber’s desire to keep its workforce individually isolated from each other and the company itself by legal actions and creating the impression that the company is inaccessible [25]. However, isolation as a coping family means that “the workers keep their suffering (from lack of care) hidden from others to protect themselves from more suffering”. This coping strategy is prevalent among adults [16], and we believe this family of coping could manifest itself in a limited set of actions, such as never complaining in forums, never joining forums or communities, or having an obsessive orientation toward staying away from clients or coworkers. Identification of coping families triggered by a threat to relatedness is difficult (as is the case for “delegation”), because, in them, the workers try to avoid interactions with context and either completely isolate themselves or just rely on their close and loved ones (i.e., “delegation”). Thus, these workers are less likely to post on forums or voice their allegations, making it hard to find such “coping instances” in data.

## 7. Limitations and Implications

This study is limited, as it is only an introduction to the motivational action theory of coping in the gig work context and is based on a selective list of literature to show the relevance of the theory to the context. The theory needs to be refined, as our investigation of some families of coping (e.g., delegation) resulted in questions rather than answers. This paper extends the literature theoretically and methodologically. Theoretically, it shows that self-determination theory can be a unifying theory for the study of gig workers’ experience and their well-being, due to its explanation power at different levels of analysis, from work characteristics to intrinsic psychological transactions and consequent coping regulations linked to both our basic psychological needs and evolutionary adaptive processes. Methodologically, it introduces an inductively and deductively generated categorization system for the study of coping that can be used in multiple methodological approaches. It can help qualitative researchers in generating coding schemes for data analysis, especially in the analysis of online forum comments. Future quantitative studies can also test the theory both from a variable-centered view and study predictors and consequent coping regulations with need frustration through empirically tested measures of these constructs (see [16]) borrowed from the gig-work context. It can also be beneficial to further our understanding of this phenomenon from a person-centered view and studying profiles of needs and coping families among workers. The Radar can also be used in quasi-experimental methods. For instance, interventions for the improvement of well-being can be tested on separate groups of people with different families of coping. The main propositions of the Radar (e.g., behaviors expected from workers when their basic psychological needs are depleted) can be examined by comparing groups of gig workers based on their platform. For instance, do the resources in one platform (e.g., chatbot) lead to different outcomes in workers compared to a group of workers who use platforms that lack these resources? The literature suggests that gig workers’ behaviors seem to have a profile of different coping families, as all three needs are being exploited at the same time for workers (bundles of control [34]), and some coping families are prerequisites for others. For instance, a “sense-making, information seeking, or studying algorithm” seems to accompany multiple other constructive (e.g., “problem solving;” “negotiation”) and rigid (e.g., “escape” or “manipulation”) families of coping. The differentiation of constructive forms of coping families from rigid forms based on their effects on well-being as challenges or threats is another promising area for future research.

The study also has important practical implications. The rhetoric of gig work is mostly concerned with debates about its benefits and threats to the work contexts and solutions that are compatible with the environment and expectations of work [52]. Our Radar categorization system shows that workers demonstrate different behaviors and emotions based on the level of risk that they perceive. Prevalence of perceptions of threat (rather than challenge) among workers implies risky working conditions that would need intervention. A focus on the satisfaction of workers’ basic psychological needs, protecting workers’ needs by law, and need supportive work designs are key interventions that may improve the psychological health of gig workers. The Radar can benefit the development of HRM practices in gig organizations and appropriate working conditions to extend workers’ capabilities and motivate them toward constructive behaviors. Developing integrated practices of recruitment, performance management, motivation, incentives, and compensation systems that foster need satisfaction and provide the resources for constructive forms of coping families can help organizations protect workers’ well-being and gain long-term success. For instance, Jabagi et al. [1] show that integration of enterprise social media (ESM) in the platform can lead to intrinsic motivation through the satisfaction of relatedness needs. In radar terms, these tools can give gig workers resources to show “support seeking behavior” and decrease the likelihood of “isolation”. Similarly, empowering employees with tools or resources for finding information about their work and rights can support the need for competence and increase the likelihood of “information seeking” and “problem-solving” behaviors rather than “escape” or “helplessness”. In the same way, integrating the possibility of negotiations with clients can decrease the likelihood of “opposition” and “destructive behavior at work.

## 8. Conclusions

Research on coping with stress is crucial, especially in the context of gig work, where the arrangements of the work itself seem to be a source of mild to extreme levels of stress and may contribute to worker ill-being. Lack of consensus on coping can impede the development of the field, theoretically and methodologically, and this is extremely important for the immature studies of the gig-worker context. Our exhaustive and comprehensive framework of coping families borrowed from Skinner et al. [16,18] includes multiple dimensions of coping that have the potential to explain all types of coping behaviors and theoretically differentiate or connect them to each other, to individuals’ basic and inner psychological needs, and to their adaptation to the environment. Besides being exhaustive and complex, it is parsimonious and coherent in the sense that it is centered on three basic psychological needs, and these needs manifest themselves both in predictors of coping (contextual demands) and in the categorization of ways of coping with higher-order categories linking them to adaptive processes.

## Figures and Tables

**Table 1:** Predictors of frustration of basic psychological needs in academic and gig-work contexts.

Context	Autonomy	Competence	Relatedness
Academic Context: Children and Adults [16,18]	Pressures by rules or rewards to direct behaviors, emotions, goals, and actions	Noncontingent, Inconsistent, Discriminatory, Unfair contexts Lack of information about how to produce desired effects	Lack of care, respect, communication, and affection from important others
Gig work Context Adult Workers [3,12]	Incentives and Pressures for a high gig acceptance rate	Lack of positive or constructive feedback from the algorithm	Isolation from Coworkers
	Financial Dependency on Platform	Lack of career mentor	Lack of well-being support
	Controls on pay rate	Algorithmic one-way rating systems and corresponding penalizations	Isolation from family/friends because of overwork

**Table 2:** Families of coping identified in gig-worker coping literature.

Need	Coping Family	Appraisal Pattern	Bucher et al. [23]	Muszy̤ski et al. [30]	Pregenzer et al. [31]	Jarrahi and Sutherl [13]
Autonomy	Submission	I don’t know what I want.				
	Accommodation	I will decide.	Keeping Emotions in check	Loyalty and voice	Blending (Embracing control, Success reports, Exerting effort to satisfy customers, Debunking allegations)	
	Negotiation	I will reduce pressure.	Keeping Emotions in check	Loyalty and voice		
	Opposition	The world is hostile.		Exiting	Separating (Voicing allegations, Resentment, Quitting, Destructive behaviors)	
Competence	Helplessness	I cannot accomplish this.	Undervaluing Work		Bridging (Wishful thinking)	
	Problem Solving	I can accomplish.				Manipulating the algorithm
	Information Seeking	I will learn.				Sense-making
	Escape	The world is unfair.	Staying under the radar Curtaining client outreach		Distancing (Complaining, Avoiding control, Sarcasm, Gaming)	Circumventing
Relatedness	Delegation	I am alone.				
	Self–Reliance	I will connect.		Loyalty [without voice]		
	Support Seeking	I will reduce separation.			Separating (Voicing allegations)	
	Isolation	The world is cold.				

## Data Availability

Not applicable.
